# Kommerell's Diverticulum: An Unusual Cause of Chronic Cough

**DOI:** 10.1155/2012/512790

**Published:** 2012-11-20

**Authors:** Rahul Magazine, Charudutt Sambhaji, Ranjan Shetty, Umesh Goneppanavar

**Affiliations:** ^1^Department of Pulmonary Medicine, Kasturba Hospital, Kasturba Medical College, Manipal University, Manipal 576 104, India; ^2^Department of Radiodiagnosis and Imaging, Kasturba Hospital, Kasturba Medical College, Manipal University, Manipal 576 104, India; ^3^Department of Cardiology, Kasturba Hospital, Kasturba Medical College, Manipal University, Manipal 576 104, India; ^4^Department of Anaesthesia, Kasturba Hospital, Kasturba Medical College, Manipal University, Manipal 576 104, India

## Abstract

A 62-year-old male presented to the outpatient department of chest with history of dry cough since two months and swelling on the anterior aspect of neck of 30-year duration. Physical examination revealed a goitre. However, further imaging studies revealed presence of another associated pathology, a Kommerell's diverticulum in association with a right aortic arch with aberrant left subclavian artery. The enlarged thyroid was not compressing the trachea, and its occurrence in this case could be incidental. The diverticulum was considered as the cause of chronic cough in our case as it was causing tracheal compression, and also there were no other obvious causes which could explain the symptom. Vascular anomalies such as Kommerell's diverticulum, though uncommon, should be considered in the differential diagnosis of chronic cough particularly when other common causes have been ruled out.

## 1. Introduction

Burckhard Kommerell described an aortic diverticulum for the first time in 1936 in a living patient [1]. This saccular aneurysmal dilation at the origin of aberrant subclavian artery consists of an aneurysm of the thoracic aorta as well as an aneurysmal opening of an aberrant subclavian artery [2, 3]. It is an uncommon condition that occurs in association with a left aortic arch with aberrant right subclavian artery (prevalence of 0.5%–2.0%), or a right aortic arch with aberrant left subclavian artery (0.05%–0.1%) [3]. We present one such case of Kommerell's diverticulum that presented in an unusual way. Symptomatic Kommerell's diverticulum usually manifests with chest symptoms or dysphagia. In this particular case, the patient presented with an uncommon manifestation in the form of chronic cough.

## 2. Case Report

**Figure 1:**
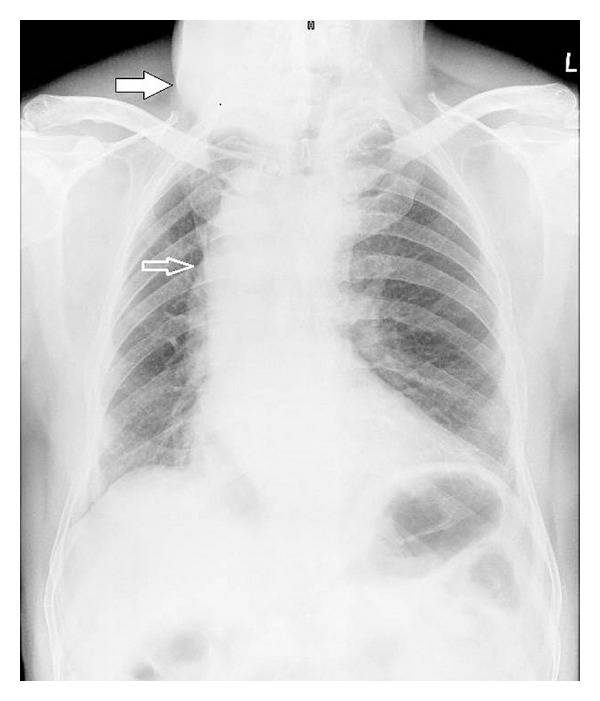
Frontal chest radiograph shows evidence of right-sided aortic arch (open arrow) and also a soft-tissue density in the neck representing thyromegaly (solid arrow).

**Figure 2:**
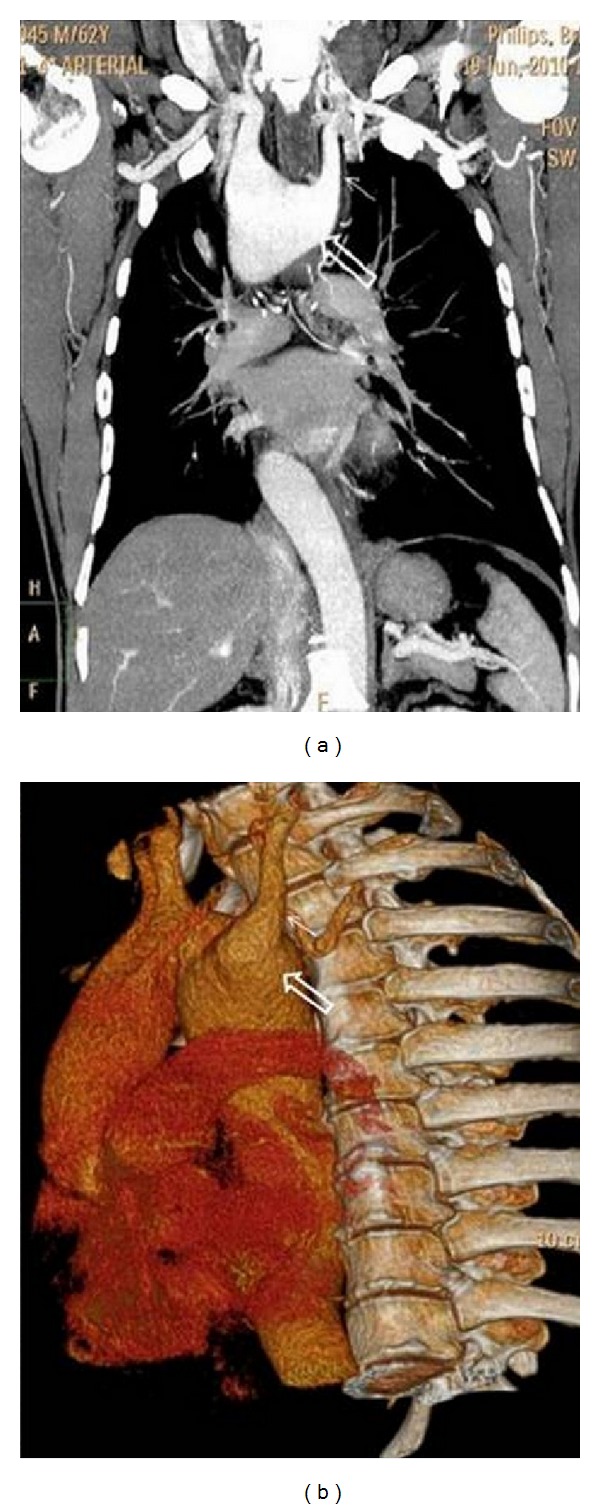
Coronal reconstruction (a) and volume-rendered (b) images of thorax demonstrate right-sided aortic arch with aberrant left subclavian artery (arrow) showing Kommerell's diverticulum (open arrow) at its origin.

**Figure 3:**
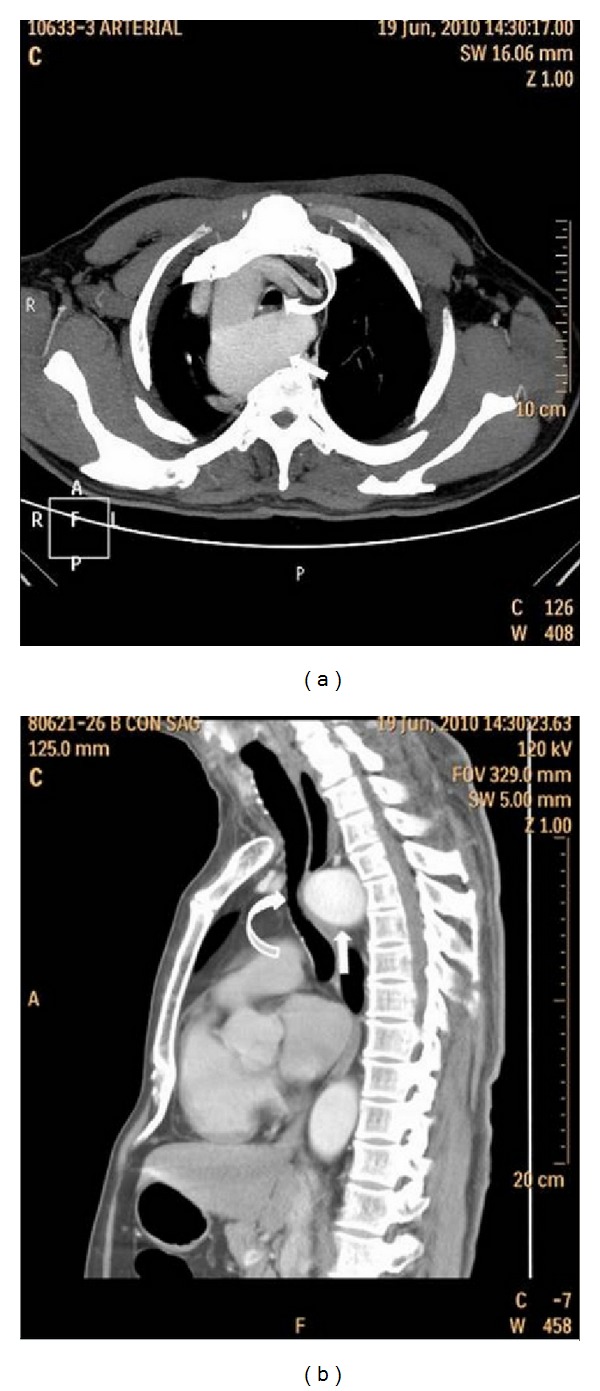
Contrast-enhanced computerized tomogram of thorax demonstrating trachea (curved solid arrow) narrowed between the arch of the aorta and the Kommerell's diverticulum (arrow).

A 62-year-old man presented to the outpatient department of chest with history of insidious onset of dry cough of two-month duration. He also had a swelling on the anterior aspect of his neck since the past 30 years. There was no history of stridor, breathlessness, dysphagia, nasal symptoms, heartburns, or any constitutional symptoms. Review of his medical records revealed that he had been prescribed inhaled steroids, bronchodilators, antihistaminics, and proton pump inhibitors for the treatment of his cough, but he was not relieved of his symptom. He did not smoke or consume alcohol. On general physical examination, patient was moderately built and nourished, afebrile, with a pulse rate 75/min, regular and good volume, respiratory rate 14/min, and blood pressure 128/86 mm Hg. There was a firm, nontender swelling of 8 cm × 7 cm size on the anterior aspect of the neck which moved with deglutition. The skin over the swelling was normal. There was no significant lymphadenopathy. Chest examination was normal. The otorhinolaryngological (ENT) evaluation was also normal except for the presence of diffuse enlargement of thyroid gland which was nonpulsatile. Examination of abdomen and other systems did not reveal any abnormality. The patient's serology was negative for retrovirus. Hemogram, blood biochemistry, serum electrolytes, and thyroid function tests were within normal limits. The Mantoux test showed no induration. Routine urine analysis was normal. Three induced-sputum sample smears were negative for acid fast bacilli. Fine needle aspiration and cytology of the thyroid demonstrated the presence of a colloid goitre. Pulmonary function testing did not show any evidence of reactive airway disease but instead was suggestive of variable intrathoracic airway obstruction. Frontal view of the chest roentgenogram showeda right-sided aortic archand alsothyroid enlargement (Figure 1). Computed tomography (CT) of thorax revealed (Figures 2 and 3) the presence of a right-sided aortic arch with aberrant left subclavian artery showing Kommerell's diverticulum at its origin as well as the tracheal narrowing between the arch of the aorta and the Kommerell's diverticulum. Fiberoptic bronchoscopy did not reveal any abnormality except for compression of the trachea corresponding to the site of anomaly.

## 3. Discussion

In patients with a right aortic arch, Kommerell's diverticulum is an embryologic remnant of the left fourth aortic arch posteriorly. Though the diverticulum can present with chest symptoms or dysphagia, it may not always cause symptoms [1, 3]. The enlargement of the Kommerell's diverticulum by itself and the sling-like effect of the left subclavian artery, which pulls the right aortic arch towards left side, are responsible for compression of the trachea [4]. Age-related atherosclerotic changes occurring in the diverticulum could be another mechanism that may contribute to compression of the surrounding structures [3]. In our case, Kommerell's diverticulum did not produce any symptoms till the age of 62 years, and this latter mechanism could explain the late onset of symptoms. Initially, we also considered the possibility of goitre related compression of the trachea as the cause of cough. However, CT scan of the neck revealed that the goitre did not have an intrathoracic extension and was not compressing the trachea either in the extrathoracic or in the intrathoracic part. History, physical examination, and relevant investigations were used to rule out other causes of chronic cough such as bronchial asthma, gastroesophageal reflux disease, and ENT-related problems. A case of chronic cough in a patient with aberrant right subclavian artery syndrome and Kommerell's diverticulum has been reported earlier in the literature. However, the authors could not demonstrate tracheal compression on imaging studies [5]. The diverticulum, if neglected, can lead to serious complications such as aortic rupture, dissection, or distal embolization [6]. Up to 19% of patients present with aortic rupture, and the mortality is very high in such cases [7].

Unfortunately, in our case we could not proceed ahead with surgical correction of this vascular anomaly as the patient refused to undergo any procedure. As a result we could not demonstrate with certainty the causality relationship between the anomaly and the symptoms. However, in view of the CT thorax findings and after ruling out other possible causes of chronic cough, we are fairly confident to conclude that the most probable cause of chronic cough in our case was Kommerell's-diverticulum-related compression of the trachea. This case illustrates a rare cause of chronic cough.
